# Competition between Influenza A Virus Genome Segments

**DOI:** 10.1371/journal.pone.0047529

**Published:** 2012-10-11

**Authors:** Ivy Widjaja, Erik de Vries, Peter J. M. Rottier, Cornelis A. M. de Haan

**Affiliations:** Virology Division, Department of Infectious Diseases and Immunology, Faculty of Veterinary Medicine, Utrecht University, Utrecht, The Netherlands; University of Ottawa, Canada

## Abstract

Influenza A virus (IAV) contains a segmented negative-strand RNA genome. How IAV balances the replication and transcription of its multiple genome segments is not understood. We developed a dual competition assay based on the co-transfection of firefly or *Gaussia* luciferase-encoding genome segments together with plasmids encoding IAV polymerase subunits and nucleoprotein. At limiting amounts of polymerase subunits, expression of the firefly luciferase segment was negatively affected by the presence of its *Gaussia* luciferase counterpart, indicative of competition between reporter genome segments. This competition could be relieved by increasing or decreasing the relative amounts of firefly or *Gaussia* reporter segment, respectively. The balance between the luciferase expression levels was also affected by the identity of the untranslated regions (UTRs) as well as segment length. In general it appeared that genome segments displaying inherent higher expression levels were more efficient competitors of another segment. When natural genome segments were tested for their ability to suppress reporter gene expression, shorter genome segments generally reduced firefly luciferase expression to a larger extent, with the M and NS segments having the largest effect. The balance between different reporter segments was most dramatically affected by the introduction of UTR panhandle-stabilizing mutations. Furthermore, only reporter genome segments carrying these mutations were able to efficiently compete with the natural genome segments in infected cells. Our data indicate that IAV genome segments compete for available polymerases. Competition is affected by segment length, coding region, and UTRs. This competition is probably most apparent early during infection, when limiting amounts of polymerases are present, and may contribute to the regulation of segment-specific replication and transcription.

## Introduction

The mechanism of replication and transcription varies greatly among viruses depending on the nature and structure of their viral genomes. Negative-strand RNA viruses replicate their viral genome via the synthesis of full length positive-strand complementary RNA (cRNA) molecules that in turn serve as templates for the synthesis of negative-strand virion RNA (vRNA) genomes. The negative-strand genomes also function as templates for the production of mRNAs [1], [2]. In non-segmented negative-strand RNA viruses, sequential transcription of successive genes results in a gradient of transcript abundance that steadily decreases towards the end of the template. Thus, the expression level of each gene is governed by the gene order [3]. This does, however, not apply to all negative-strand viruses as some of them acquired segmented genomes during their evolution. Each genome segment of these viruses is individually replicated and transcribed, necessitating careful regulation of these distinctive processes to generate sufficient vRNAs and proteins for the production of progeny virions [2].

Influenza A virus (IAV) of the family *Orthomyxoviridae* is an enveloped, negative-strand RNA virus. The IAV genome is composed of eight different vRNA segments that altogether encode up to 13 proteins [4]–[7]. Each vRNA and cRNA possesses untranslated regions (UTRs) of varying length at the 3′ and 5′ ends. The first 12 and 13 nucleotides at the 3′ and 5′ UTRs of the vRNAs and cRNAs are highly conserved among different RNA segments. These highly conserved partly complementary UTRs, which form a “panhandle” or “corkscrew” conformation by alternative modes of base-pairing, constitute the promoter structure for RNA synthesis [8], [9]. The panhandle conformation results from base-pairing between 5′ and 3′ terminal ends of the viral RNA segment with a small internal loop [10], [11], while the corkscrew structure consists of a six base-pair RNA rod in the distal element in conjunction with two stem–loop structures of two short-range base-pairs [12].

**Figure 1:**
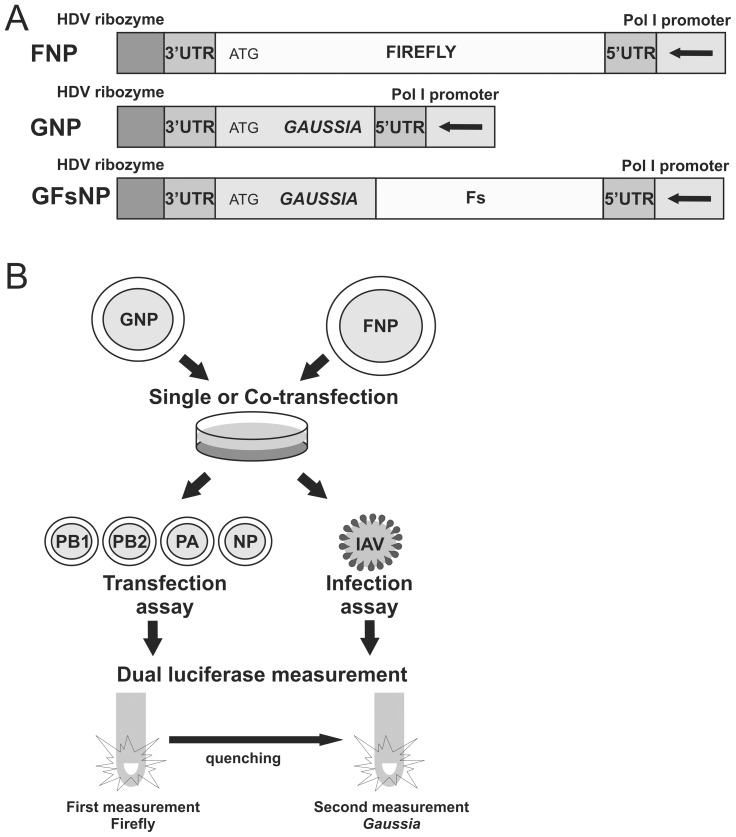
Schematic representations of the dual luciferase reporter constructs, and of transfection and infection assays. A) Schematic outline of the firefly and *Gaussia* luciferase reporter constructs. The firefly and *Gaussia* luciferase genes, flanked by 3′ and 5′ UTR of the NP segment, were inserted in antisense orientation between a PolI promoter and a ribozyme sequence, resulting in FNP and GNP, respectively. The extended *Gaussia* luciferase reporter construct (GFsNP) additionally contains the 3′ terminal half of the firefly luciferase gene (indicated as Fs) behind the stop codon of the *Gaussia* gene. B) HEK 293T cells were transfected with one or both reporter constructs (single or co-transfection). Luciferase expression is induced by expression of viral RNA polymerases (PB1, PB2, PA) and NP either by simultaneous co-transfection of expression plasmids (transfection assay) or by infection with IAV at an MOI 1 at 24 h post-transfection (infection assay). The firefly and *Gaussia* luciferase expression levels can be measured consecutively using a dual luciferase assay system (Promega) 24 h post-transfection or post-infection.

The IAV vRNA and cRNA segments form ribonucleoprotein (RNP) complexes by association to the polymerase and to multiple copies of the nucleoprotein (NP). These RNPs may be regarded as independent molecular machines responsible for transcription and replication of each segment. The viral RNA polymerase, which consists of the PA, PB1 and PB2 subunits, recognizes the RNA promoter, and stabilizes a supercoiled conformation of the RNPs. Different models have been proposed for the regulation of transcription and replication. One model suggests that the RNA polymerase switches from a transcriptase, used for mRNA synthesis, to a replicase, used for cRNA and vRNA synthesis, which is triggered by newly synthesized NP protein [13]. Another model suggests that cRNAs can be directly synthesized from incoming vRNAs, but require newly synthesized polymerase and NP to be stabilized in RNPs [14]. More recently, Jorba and colleagues proposed a model, in which a template RNP is replicated *in trans* by a soluble polymerase complex, whereas transcription of the vRNA occurs *in cis* and the resident polymerase complex is responsible for mRNA synthesis [15].

**Figure 2:**
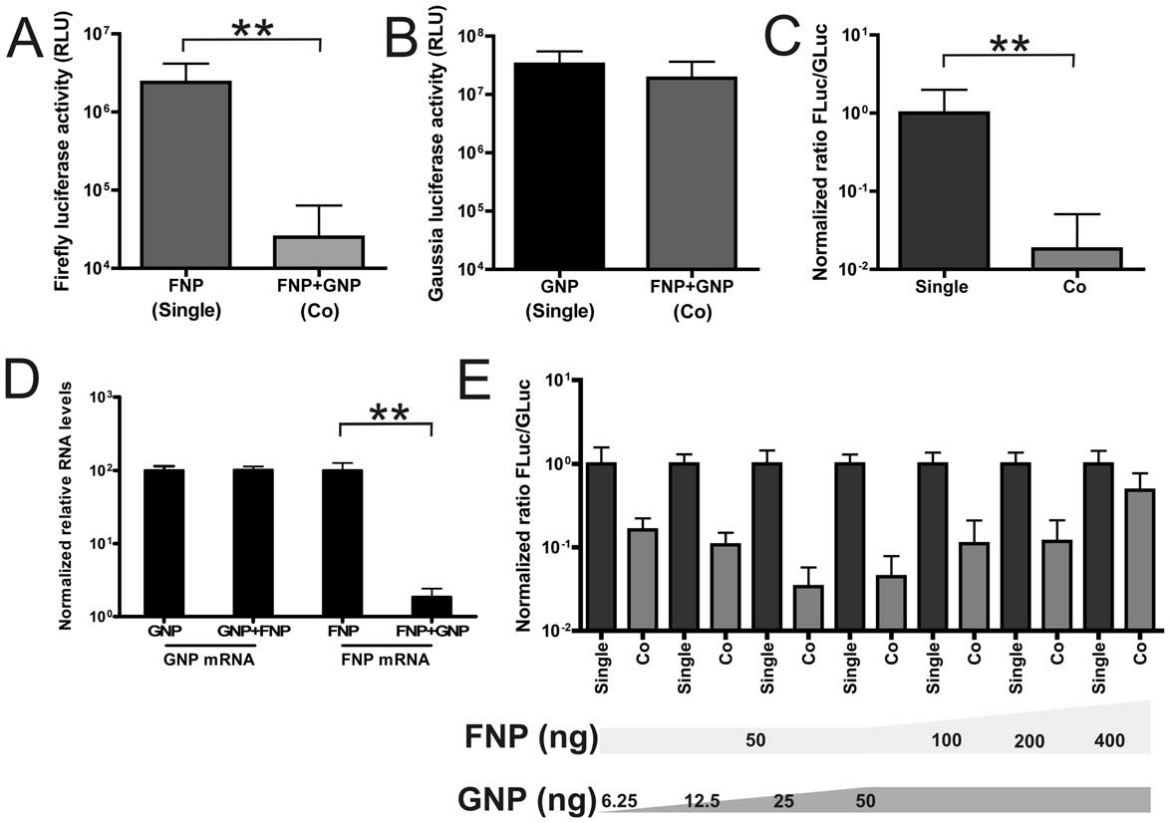
Competition between firefly and *Gaussia* luciferase reporter genome segments. Plasmids encoding firefly (FNP) or *Gaussia* (GNP) luciferase reporter constructs were transfected alone (Single) or in combination (Co). Luciferase expression was induced by simultaneous co-transfection of polymerase and NP expression plasmids (transfection assay). A) Firefly luciferase activity after transfection of FNP or FNP together with GNP. B) *Gaussia* luciferase activity after transfection of GNP or GNP together with FNP. C) Normalized ratio of firefly to *Gaussia* luciferase activity (Fluc/Gluc) when FNP and GNP were transfected singly or in combination. D) Quantitative RT-PCR analysis of mRNA levels derived from FNP and GNP after single or co-transfection of these constructs. RNAs were extracted 24 h post-transfection and subjected to quantitative RT-PCR. The comparative Ct method was used to determine the relative mRNA levels using the housekeeping gene GAPDH as a reference. The mRNA levels were normalized relative to the samples in which a single reporter construct was transfected. E) Normalized ratio of firefly to *Gaussia* luciferase activity (Fluc/Gluc) when reporter gene constructs FNP and GNP were transfected singly or in combination. The amounts of reporter gene constructs transfected are indicated. Significant differences in A–D are indicated (**; P<0,01).

Early studies, in which semi-quantitative hybridization techniques were used, described differential expression rates and levels of the different vRNAs. In general it appeared that the mRNAs for NS1 and NP are synthesized preferentially at the early times post infection, while the synthesis of matrix (M1) mRNA is delayed [16]–[18]. More recently, Vester and coworkers showed, by using quantitative RT-PCR that the vRNAs are synthesized in equimolar amounts and with similar kinetics, whereas early in infection preferential synthesis of NS1 mRNA and a delay in that of M1 mRNA was found [19]. However, how IAV temporally regulates the replication and transcription levels of its multiple genome segments is not known.

**Figure 3:**
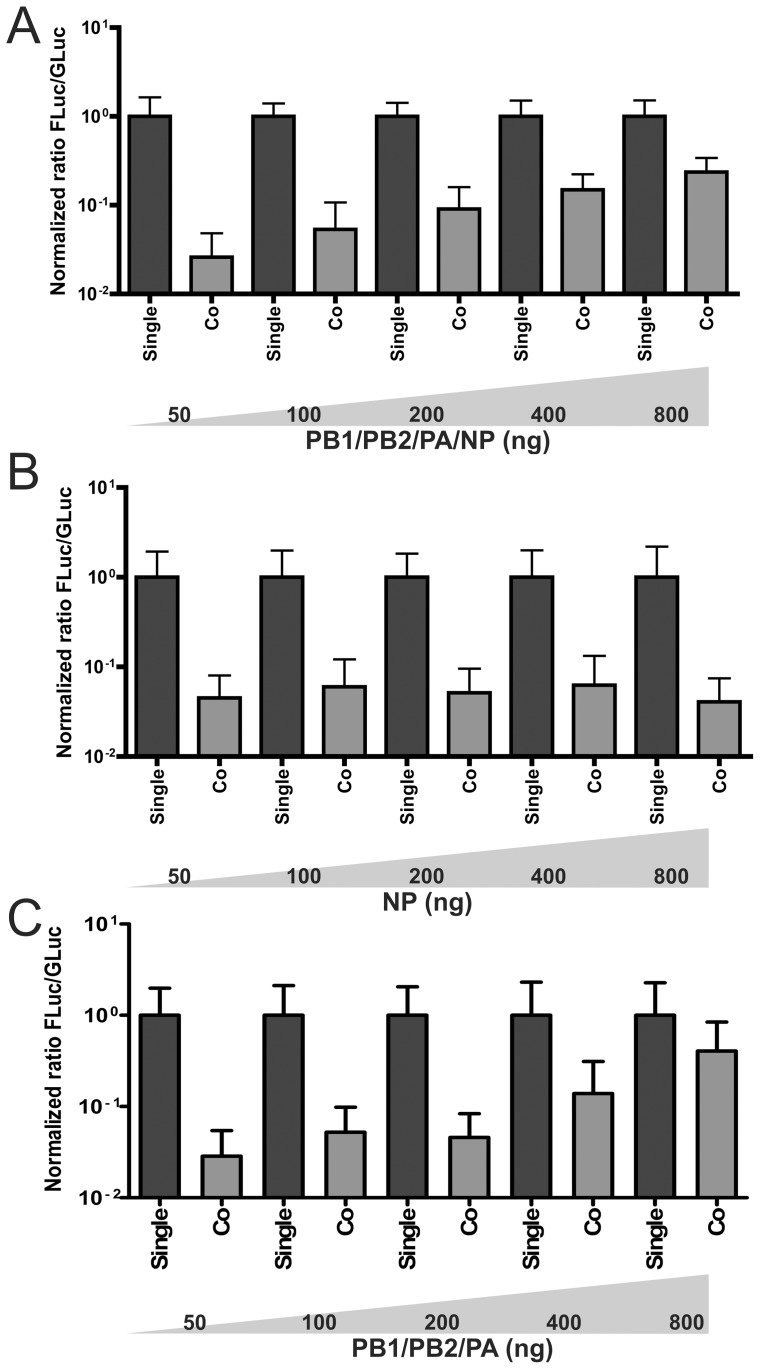
Competition for viral proteins. Normalized ratio of firefly to *Gaussia* luciferase activity (Fluc/Gluc) after single (Single) or co-transfection (Co) of FNP and GNP in the presence of increasing amounts of transfected plasmids encoding PB1, PB2, PA and NP (A), NP alone (B) or PB1, PB2 and PA (C).

Several reporter assays have been described to study and quantify IAV transcription/replication *in vivo*. These reporter systems usually consist of a reporter protein-encoding cDNA, flanked by 3′ and 5′ UTRs, inserted in an antisense orientation between a PolI promoter and a transcription terminator or ribozyme sequence. After introduction of the reporter construct into a cell, reporter gene expression is induced by co-transfection of plasmids encoding NP, PA, PB1 and PB2 (transfection assay) or by subsequent infection with a helper IAV (infection assay). Such reporter assays are very helpful to quantify virus replication or virus production, and to assess the antiviral activity of compounds including antibodies [20]–[22]. These assays have also been used for the mutational analysis of IAV promoter elements *in vivo* [12], [23], [24].

**Figure 4:**
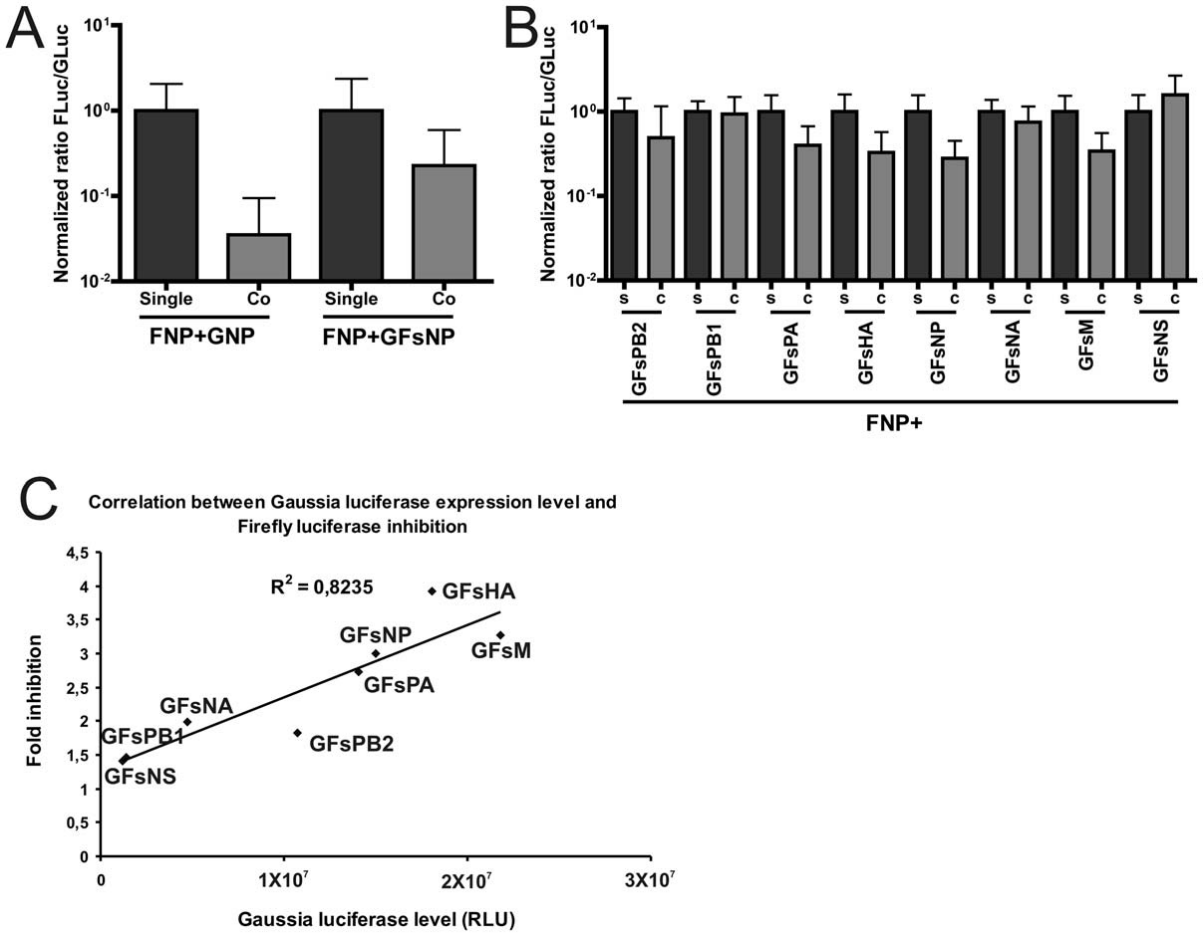
The effect of gene length and segment UTR. A) Plasmids encoding firefly (FNP) or *Gaussia* luciferase reporter constructs were transfected alone (Single; s) or in combination (Co; c). Luciferase expression was induced by simultaneous co-transfection of polymerase and NP expression plasmids (transfection assay). A) Normalized ratio of firefly to *Gaussia* luciferase activity (Fluc/Gluc) after single or co-transfection of FNP and GNP or GFsNP (extended version). B) Normalized ratio of firefly to *Gaussia* luciferase activity (Fluc/Gluc) after single or co-transfection of FNP and different versions of the extended *Gaussia* reporter construct carrying UTRs derived from the eight IAV-WSN genome segments. C) Correlation between fold inhibition of firefly luciferase expression upon co-transfection of a *Gaussia* luciferase reporter construct and the corresponding *Gaussia* luciferase expression levels after single transfection is shown.

To get more insight in the mechanism by which IAV regulates and balances the replication and transcription of its genome segments, we converted the IAV single reporter assay into a dual reporter assay, by which the expression of two different luciferase genes can be monitored simultaneously. This assay more closely resembles the multiple segment transcription and replication conditions that occur in IAV infected cells than the single reporter assay. Our results indicate that different vRNA segments compete with each other, as transcription/replication of one vRNA segment can affect that of another. Using this multiple segment reporter assay we subsequently assessed the contribution of vRNA segment length, UTRs sequence and coding sequence to the competition between the different segments.

**Table 1:** vRNA segment lengths and UTR sequences of IAV-WSN used in the reporter constructs.

Segment	Length	3′UTR sequence^a^	5′UTR sequence^a^
PB2	2280	UCGCUUUCGUCCAGUUAAUAUAAGUUA *UAC*	*AUC* ACAGCUUAUCAAAUUUUUGCUGGAACAAAGAUGA
PB1	2274	UCGCUUUCGUCCGUUUGGUAAACU *UAC*	*AUC* ACUUAAAUCGAACAGGAAGUACUUUUUUACGGAACAAAGAUGA
PA	2151	UCGCUUUCGUCCAUGACUAAGUUU *UAC*	*AUC* AACACCGUUACGAUGAUAAACGAUAGGUAUGACA GGUUUUUUCAUGGAACAAAGAUGA
HA	1698	UCGUUUUCGUCCCCUUUUAUUUUUG UUGGUUU *UAC*	*ACU* CUAAUCCUAAAGUCUUUAUAUUCCUUUUUGU GGGAACAAAGAUGA
NP	1497	UCGUUUUCGUCCCAUCUAUUAGUGA GUGUCUCACUGUAGCUUUAG *UAC*	*AUU* UCUUUUUAUGGGAACAAAGAUGA
NPph	1497	UCAUCUUUGUCCCAUCUAUUAGUGA GUGACUCACUGUAGCCA *UAC*	*AUU* UCUUUUUAUGGGAACAAAGAUGA
NPphR	1497	UCGUUUUCGUCCCAUCUAUUAGUGA GUGUCUCACUGUAGCUUUAG *UAC*	*AUU* UCUUUUUAUGGGAACGAAAACGA
NA	1362	UCGCUUUCGUCCUCAAAUU *UAC*	*AUC* AAACAAGUUUUUUGAGGAACAAAGAUGA
M	759	UCGUUUUCGUCCAUCUAUAACUUUC *UAC*	*AUU* UUUUGAUGGAACAAAGAUGA
NS	693	UCGUUUUCGUCCCACUGUUUCUGUAU *UAC*	*AUU* AUUAUUUUUUGUGGGAACAAAGAUGA

a The conserved regions in the 3′ and 5′ UTRs are underlined. The start and stop codons are italicized. The bold characters indicate the mutated nucleotides.

## Results

### Dual luciferase assay to study IAV transcription/replication

A schematic overview of the dual luciferase reporter constructs and the assays is shown in Figure 1. The firefly and *Gaussia* luciferase genes, in this example flanked by 3′ and 5′ UTRs of the NP segment (referred to as FNP and GNP, respectively), are inserted in an antisense orientation between a PolI promoter and a ribozyme sequence. Cells are transfected with either one or both reporter constructs (single or co-transfection). Luciferase expression is induced by expression of viral RNA polymerases and NP either by co-transfection of expression plasmids (transfection assay) or by virus infection (infection assay). The expression levels of the firefly and *Gaussia* luciferase reporter constructs are determined consecutively using a single tube, dual luciferase assay system.

### Competition between reporter segments

First we determined the luciferase expression levels of the firefly (FNP) and *Gaussia* (GNP) luciferase reporter constructs when transfected alone or in combination by using the transfection assay. As shown in Figure 2, single transfection of each reporter gene resulted in high expression levels of both the firefly (Fig. 2A) and *Gaussia* (Fig. 2B) luciferase genes. However, when the reporter constructs were co-transfected, the firefly luciferase expression level was dramatically reduced (Fig. 2A), while the *Gaussia* luciferase expression level in the same cells was not affected when compared to the single-transfected cells (Fig. 2B). The differential expression of firefly and *Gaussia* reporter plasmids when transfected alone or together can also be illustrated by plotting the normalized ratio of firefly to *Gaussia* luciferase activity (normFluc/Gluc; the normalized ratio's were calculated as indicated in the Materials and Methods section). As shown in Figure 2C, this ratio is significantly decreased upon co-transfection of the two reporter constructs, when compared to the ratio of the luciferase expression levels in the single-transfected cells. Similar results were obtained at earlier and later time points post transfection (data not shown). This indicates that the balance of firefly and *Gaussia* luciferase expression is strongly in favor of *Gaussia* luciferase, when both reporter constructs are present within the same cell. Thus, the results indicate that expression of the firefly luciferase gene is negatively affected by co-transfection of the *Gaussia* luciferase reporter plasmid.

Very similar results were obtained when an empty plasmid (pUC18) was included in the transfection mixture when only one reporter construct was transfected (Fig. S1A–C). Thus, the observed differences in firefly luciferase expression do not result from a lower transfection efficiency of the firefly luciferase, but not of the *Gaussia* luciferase reporter construct, when an additional plasmid was included in the transfection mixture.

Next, to analyze whether the observed difference in luciferase protein levels results from differences at the RNA level, we performed a quantitative RT-PCR analysis of the mRNA levels [19]. The results are shown in Figure 2D. The mRNA levels of the *Gaussia* reporter gene were not affected by co-transfection of the other reporter construct. However, co-transfection of the *Gaussia* luciferase construct significantly affected mRNA levels of the firefly luciferase gene. From these results we conclude that the observed inhibitory effect of co-transfection of the *Gaussia* luciferase construct on the firefly luciferase activity is a reflection of lower firefly luciferase mRNA levels.

The results indicate that replication and transcription of the *Gaussia* and firefly luciferase genome segments are in competition with each other. If so the observed inhibitory effect of co-transfection of the *Gaussia* luciferase construct on the firefly luciferase expression level is expected to depend on the ratio of the transfected reporter constructs. As shown in Figure 2E, this is indeed the case. Lowering the amount of co-transfected *Gaussia* as well as increasing the amount of co-transfected firefly luciferase reporter plasmid shifted the balance in the competition between the firefly and *Gaussia* luciferase genome segments as judged from the increased normFluc/Gluc ratio (Fig. 2E). This increased ratio resulted from altered firefly rather than *Gaussia* luciferase expression levels (Fig. S2A and B). From these results we conclude that the *Gaussia* luciferase genome segment is much more efficiently replicated and transcribed than its firefly luciferase counterpart, the latter of which is outcompeted by the presence of the former.

### Competition for the viral proteins

The firefly and *Gaussia* luciferase genome segments are most likely competing for host and/or viral factors that are necessary for transcription and/or replication. To analyze whether a limiting availability of viral proteins is an important factor in the competition between firefly and *Gaussia* luciferase genome segments, we increased the amount of plasmids encoding the RNA polymerase subunits and NP in the transfection assay. Empty plasmid (pUC18) was included in the transfection mixture when needed to achieve the same total amount plasmid DNA for each transfection condition. Upon increasing the amounts of transfected plasmids encoding PB1/PB2/PA/NP, the normFluc/Gluc ratio increased ∼10-fold (Fig. 3A) as a result of increased firefly luciferase expression levels (Fig. S3A and B). This result indicates that increased amounts of polymerase subunits and NP can alleviate the competition between the firefly and *Gaussia* luciferase genome segments. Increasing the amount of transfected NP-encoding plasmid alone did not affect the normFluc/Gluc ratio (Fig. 3B) or the absolute Fluc and Gluc levels (Fig. S3C and D). Increasing the amount of polymerase subunit-encoding plasmids, but not of the NP-encoding plasmid, appeared to alleviate the competition between the two segments (Fig. 3C). However, it also negatively affected the reporter gene expression levels *per se*, with most dramatic effects being observed for the firefly luciferase reporter segment (Fig. S3E and F). We speculate that this negative effect correlates with the requirement for NP for replication, which appears less stringent for short RNA templates [25], [26]. Although we did not analyze the NP and polymerase protein levels directly, our results indicate that the luciferase genome segments compete for RNA polymerase subunits and/or NP, with a limiting amount of polymerase subunits being the most likely explanation for the observed competition. However, we cannot exclude that the observed competition between reporter segments is partly caused by limiting amounts of host factors.

### Genome segment properties that affect competition

Next we analyzed to what extent the competition between the luciferase genome segments is affected by characteristics of the genome segments themselves. An obvious difference between the firefly and *Gaussia* genome segments is their gene length as the firefly and *Gaussia* luciferase genes consist of 1653 and 558 nucleotides, respectively. Small genome segments are likely to be replicated faster than long ones. To test this hypothesis, we generated an extended *Gaussia* luciferase gene construct, in which part of the firefly luciferase gene (3′-terminal half) was inserted immediately behind the stop codon of the *Gaussia* luciferase gene in the GNP plasmid (referred to as GFsNP, Fig. 1A) to produce a genome segment with exactly the same length as the firefly luciferase genome segment. The extended and the normal *Gaussia* luciferase reporter segments were compared for their ability to compete with the firefly luciferase reporter segment. The absolute *Gaussia* luciferase expression level from the GFsNP segment was lower than that from the GNP segment (Fig. S4B), probably resulting from its extended length, for which is corrected by plotting normalized Fluc/Gluc ratios in Figure 4. These normFluc/Gluc ratios (Fig. 4A) indicate that the extended GFsNP segment is still a strong competitor of the FNP segment, although much less efficient than the smaller GNP segment. We conclude that segment size is an important factor in the competition between vRNA segments. However, our results also suggest that coding regions are important as FNP and GFsNP segments do not differ in size, while they contain identical 5′ and 3′UTRs.

Next, we analyzed to what extent the competition between different reporter constructs is affected by the identity of the 3′ and 5′ UTRs. The genome segment UTRs provide signals for viral RNA transcription and replication, as well as for packaging of vRNP into virus particles [27]. The first 12 and 13 nucleotides at the 3′ and 5′ UTRs, which are highly conserved among the eight viral RNA segments, constitute the promoter structure for RNA synthesis [8], [9]. Also the non-conserved regions of the different segments have been implicated in viral RNA replication [28]. We inserted the 3′ and 5′ UTRs of the eight IAV-WSN segments (Table 1) into the extended *Gaussia* reporter plasmid and tested them in the competition assay with the firefly luciferase reporter construct FNP. The extended *Gaussia* construct was chosen instead of the short version as the former construct affects expression of the firefly luciferase construct less than the latter, resulting in a more balanced system in which it will be easier to detect UTR-dependent up and down effects. Introduction of the UTRs of different segments into the extended *Gaussia* reporter construct affected the balance between the *Gaussia* and firefly luciferase expression to different extents (Fig. 4B). Introduction of PB1, NA or NS segment UTRs resulted in balanced firefly and *Gaussia* luciferase expression levels as similar ratio's were observed when expressed alone or in combination (normFluc/Gluc ∼1; Fig. 4B). When the extended *Gaussia* construct was provided with the PB2, PA, HA and M segment UTRs, the normalized ratio's observed after co-transfection with FNP were similar to those observed when the construct containing the NP segment UTRs was used (normFluc/Gluc <1), indicating that the balance had shifted in favor of *Gaussia* luciferase expression. Subsequently, we analyzed whether the absolute expression levels of the eight different *Gaussia* luciferase segments (Fig. S5A) correlated with their ability to inhibit expression of firefly luciferase (Fig. S5B). Correlation between inhibition of firefly luciferase expression and the expression of *Gaussia* luciferase with different UTRs resulted in an R^2^ value of 0.8 (Fig. 4C), which indicates that 80% of the variance in firefly luciferase inhibition correlates with variability in *Gaussia* luciferase expression levels. A similar R^2^ value was obtained when the results obtained with the short GNP reporter segment were also taken into account (Fig. S6). The results indicate that not only the expression of reporter genome segments, but also the balance between different reporter genome segments is affected by 3′ and 5′ UTRs, and that these two phenomena are largely correlated. Thus, *Gaussia* luciferase genome segments that are expressed to a higher extent are more efficient inhibitors of firefly luciferase expression driven by another genome segment.

### Competitive effect of natural IAV genome segments

**Figure 5:**
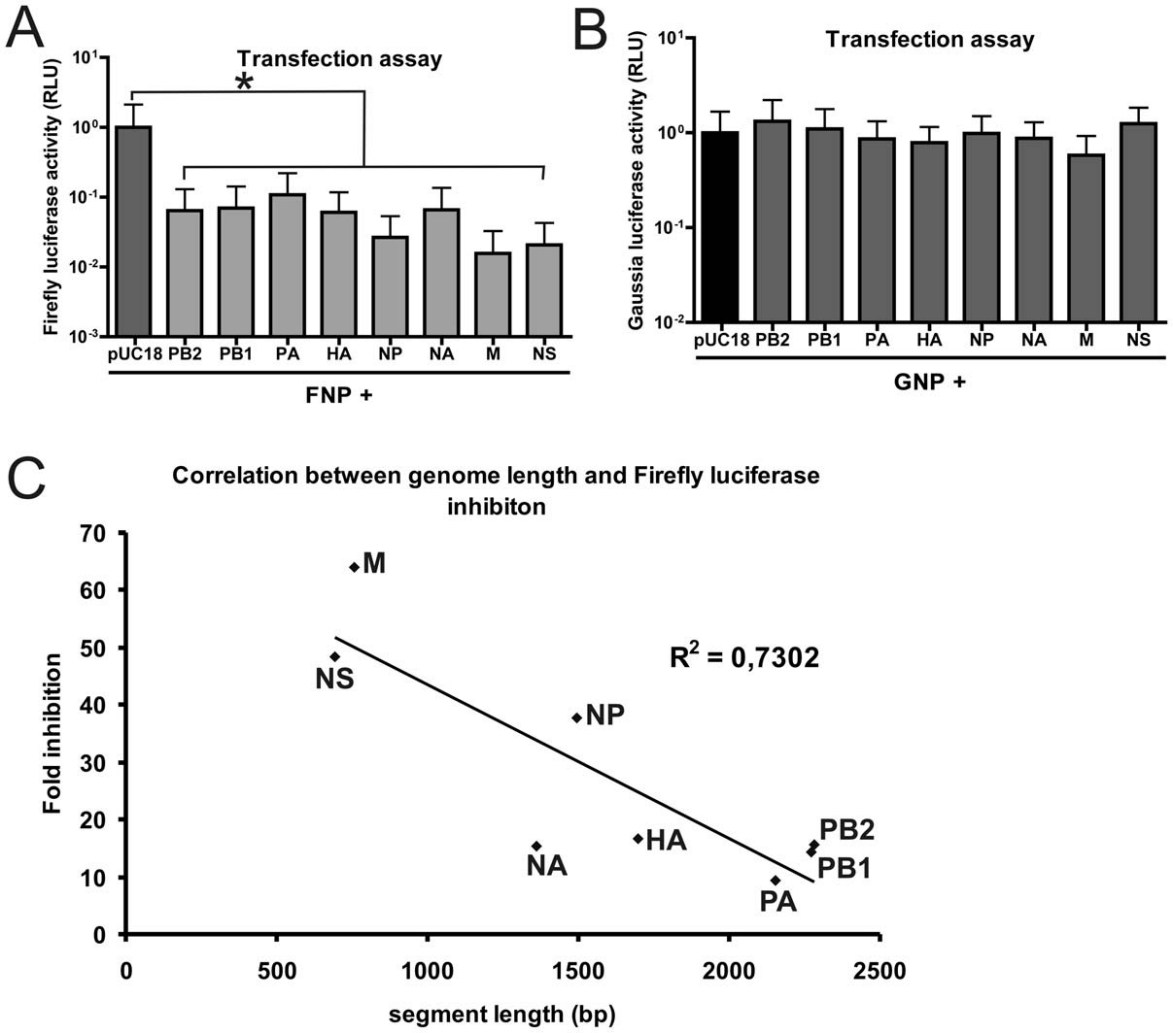
Competition between reporter and natural influenza A virus genome segments. Normalized luciferase activity of firefly (FNP) (A) or *Gaussia* (GNP) (B) luciferase reporter constructs after co-transfection with empty plasmid (pUC18) or transcription plasmids encoding one of the eight IAV-WSN vRNA segments using the transfection assay. C) Correlation between fold-inhibition of firefly luciferase activity upon co-transfection of one of the eight IAV-WSN vRNA encoding plasmids and the length of the vRNA segments. Significant differences in A and B are indicated (*; P<0,05).

Subsequently, we analyzed to what extent reporter gene expression was affected by the presence of the natural viral genome segments. To this end, the reporter constructs were co-transfected with plasmids encoding each of the IAV genome segments under the same control of human RNA polymerase I promoter. As a control, empty plasmid (pUC18) was co-transfected. The different IAV segments significantly affected the firefly luciferase levels (Fig. 5A). In general, the shorter segments gave stronger competition on the firefly expression compared to longer segments, with the M and NS segments having the largest effect. Correlation between inhibition on firefly luciferase expression and the gene length resulted in an R^2^ value of 0.7 (Fig. 5C), which indicates that 70% of the variance in firefly luciferase inhibition correlates with variability in genome segment length. In contrast, expression of *Gaussia* luciferase was hardly affected by the presence of the viral RNA segments (Fig. 5B), while no correlation was observed between the modest decrease/increase of *Gaussia* luciferase expression and the genome segment length (Fig. S7). We speculate that the lack of inhibition of *Gaussia* luciferase expression correlates with the very efficient replication/transcription of this reporter segment.

### Effect of panhandle-stabilizing mutations

**Figure 6:**
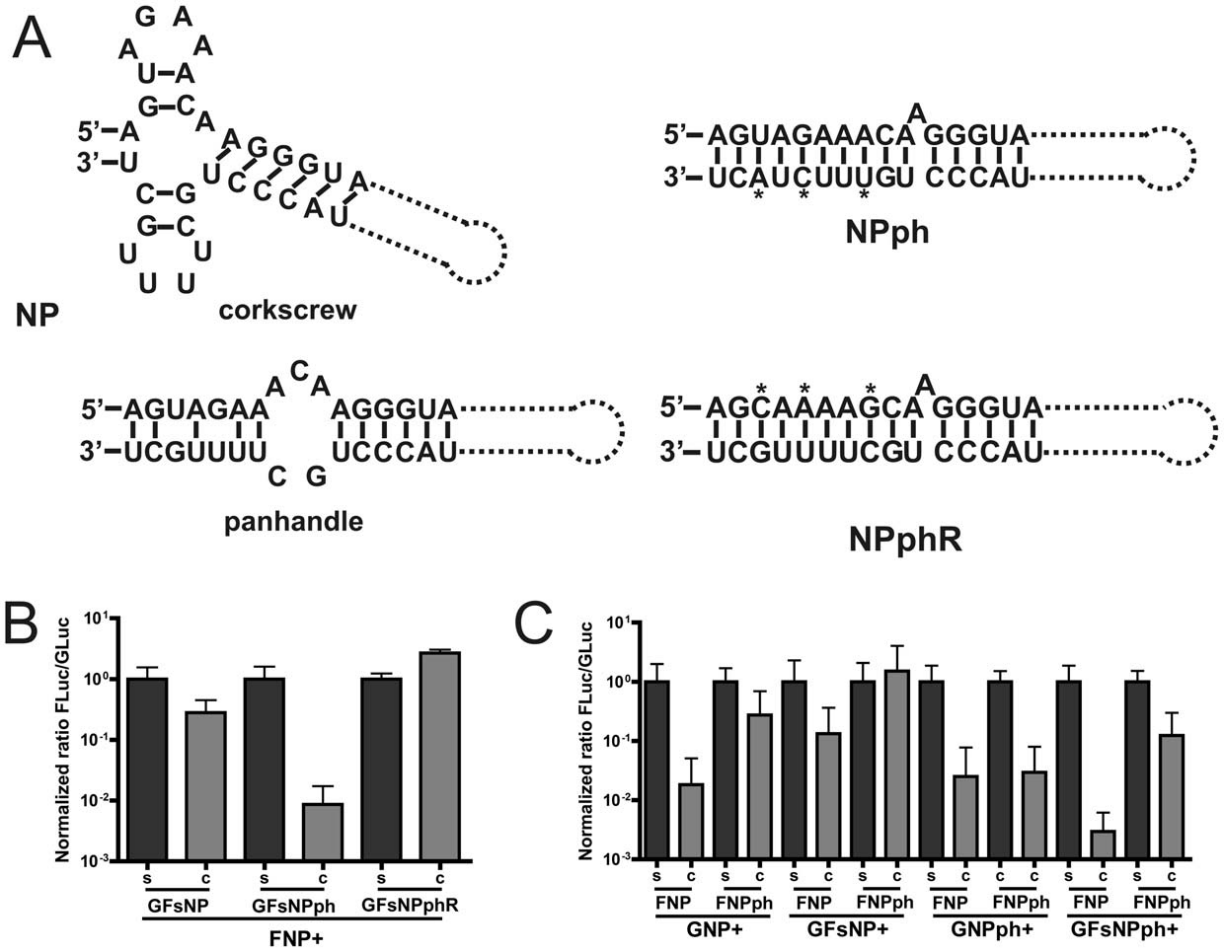
The effect of panhandle-stabilizing mutations in the UTR. A) Schematic representation of the proposed conformational structure of IAV-WSN wild type NP UTR in corkscrew or panhandle conformation (left panel; refs 10 and 30) and the improved base-pairing by panhandle-stabilizing mutations in the 3′ (NPph) or 5′ (NPphR) UTR (right panel). B) Normalized ratio of firefly to *Gaussia* luciferase activity (Fluc/Gluc) after single or co-transfection of FNP and different versions of the extended *Gaussia* reporter construct carrying either NP, NPph or NPphR UTRs (GFsNP, GFsNPph, and GFsNPphR, respectively). C) Normalized ratio of firefly to *Gaussia* luciferase activity (Fluc/Gluc) after single or co-transfection of firefly luciferase constructs with NP or NPphs UTR (FNP and FNPph, respectively) and the short or extended *Gaussia* reporter construct carrying either NP (GNP and GFsNP) or NPph (GNPph and GFsNPph) UTRs.

Mutations in the 3′UTR of the NP segment that increase gene expression have been described [23]. The nucleotide changes were predicted to improve base pairing of the 3′ and 5′ UTRs and thus to stabilize the panhandle structure. Considering the results described above, we expected that these mutations would affect competition between different reporter genome segments. The results show that reporter constructs containing these panhandle-stabilizing UTRs (referred to as NPph; Fig. 6A) indeed displayed 3–5 fold higher luciferase expression levels in the transfection assay than their wild-type UTR-containing counterparts (Fig. S8). Remarkably, however, the normalized ratio between firefly and *Gaussia* luciferase was much more affected by the presence of the NPph UTRs. Introducing the NPph UTR in the background of the extended *Gaussia* construct (GFsNPph) resulted in a much decreased normFluc/Gluc when compared to its counterpart with the wild type NP UTRs (GFsNP; Fig. 6B). A similar level of competition was not observed when the mutations that increase the number of base-pairs were introduced in the 5′ UTR of the NP segment (referred to as NPphR; Fig. 6A) instead of in the 3′UTR. In this case, the *Gaussia*, rather than the firefly luciferase expression was affected by the co-transfection of both reporter plasmids (normFluc/Gluc >1; Fig. 6B and Fig. S8B). Similar results were obtained when NPph UTR was introduced in the firefly luciferase genome segment (referred to as FNPph; Fig. 6C). In general, normFluc/Gluc was increased when FNPph rather than FNP was used, except for the combination with GNPph (Fig. 6C & Fig. S8C and D). Thus, the balance between different segments is dramatically affected by the introduction of panhandle structure-stabilizing mutations in the 3′UTR.

### Infection assay

**Figure 7:**
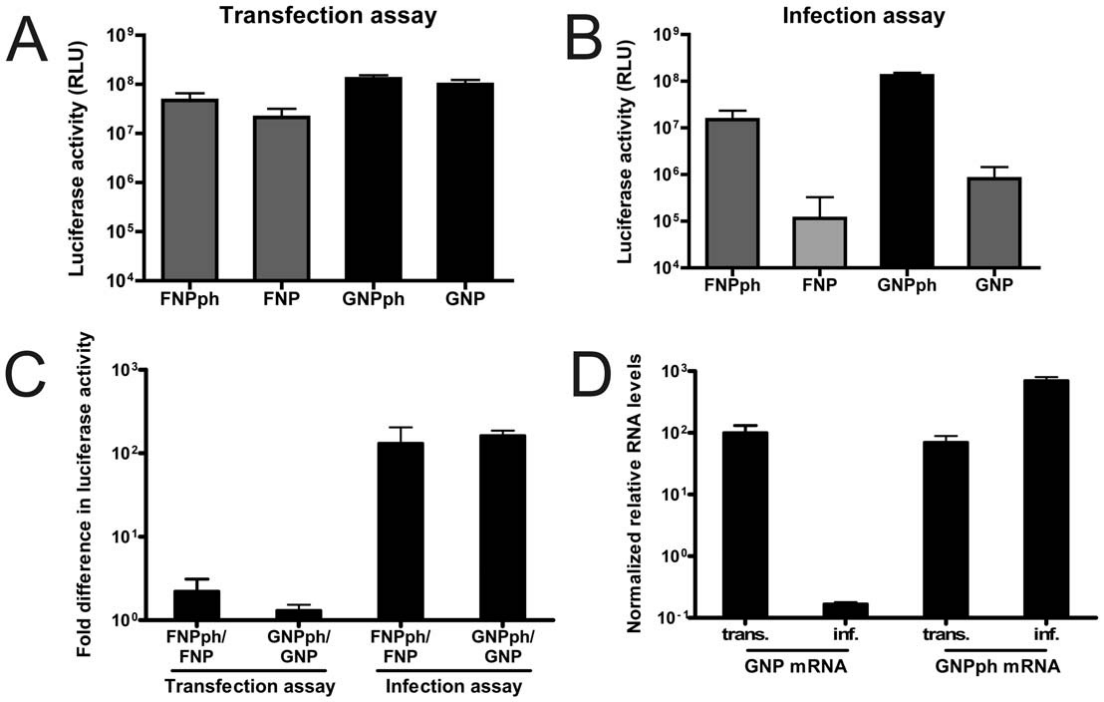
Comparison of transfection and infection assay. Luciferase activity of firefly (FNP or FNPph) or *Gaussia* (GNP or GNPph) luciferase reporter constructs using the transfection (A) or infection assay (B). B) Cells were infected with IAV-WSN at an MOI of 1 TCID50 units per cells, which resulted in approximately 50% infected cells as determined by immunocytochemical analysis using NP-specific antibodies. C) Fold difference in luciferase expression levels between FNPph and FNP and between GNPph and GNP in either the transfection or infection assay. D) Quantitative RT-PCR analysis of mRNA levels derived from GNP or GNPph using the transfection (trans) or infection (inf) assay. For the transfection assay, cells were co-transfected with expression plasmids encoding PB1, PB2, PA and NP. For the infection assay, cells transfected with reporter plasmids were infected with IAV-WSN. The comparative Ct method was used to determine the relative mRNA levels using the housekeeping gene GAPDH as a reference. The mRNA levels were normalized relative to the mRNA expression level of the GNP reporter construct in the transfection assay.

Neumann and Hobom (1995) previously reported increased reporter gene expression upon the introduction of panhandle-stabilizing mutations in the 3′ UTR. In their experimental system, however, the differences in reporter gene expression appeared much larger than ours. We hypothesized that this difference might be explained by Neuman and Hobom using virus infection to drive reporter gene expression, while we used transfection of polymerase subunit- and NP- encoding plasmids. Infection with IAV will not only provide viral RNA polymerase and NP, but will also introduce natural vRNPs that may compete with reporter genome segments for replication and/or transcription. Thus, in virus-infected cells, the natural virus genome segments might be preferentially replicated and transcribed over the reporter genome segments, unless the panhandle-stabilizing mutations in the 3′ UTR are present. To test this hypothesis we compared reporter gene expression driven by co-transfection of expression plasmids (transfection assay) with expression driven by virus infection (infection assay). Reporter genes flanked either by natural NP UTRs or by the mutant NPph UTRs were used. Both firefly and *Gaussia* luciferase genes were expressed at high levels in the transfection assay, with the reporter constructs containing the NPph UTRs again displaying somewhat higher luciferase levels than their counterparts with the natural NP UTRs (Fig. 7A). However, when using the infection assay, dramatic differences in reporter gene expression levels were observed (Fig. 7B). Thus, while the reporter genes flanked by the NPph UTRs reached 1 to 2 fold higher expression levels than those flanked by the natural NP UTR in the transfection assay, this fold difference was much increased (130 to 160 fold) in the infection assay (Fig. 7C). Quantitative RT PCR confirmed that mRNAs levels of the *Gaussia* luciferase RNAs were very similar, regardless of the presence of the natural NP UTRs or the mutant NPph UTRs in the transfection assay, but not in the infection assay (Fig. 7D). These results are in agreement with our model, in which IAV genome segments compete for available resources, likely the viral proteins, to maximize their replication and/or transcription. Only reporter genome segments carrying panhandle-stabilizing mutations in their 3′ UTR are able to efficiently compete with the natural genome segments in the infection assay.

## Discussion

The molecular mechanisms by which IAV replicates and transcribes its genome segments have generally been well studied. However, the way by which IAV regulates and balances the replication/transcription of its 8 genome segments is much less understood. In order to study and manipulate these processes, we developed a dual reporter genome segment assay that enabled us to analyze whether the replication/transcription of one genome segment is affected by that of another. Our results indicate that this is indeed the case as luciferase expression driven from a reporter genome segment was shown to be affected by the presence of other genome segments, both in the context of virus infection and in the presence of polymerase and NP proteins provided by transfection of the expression plasmids. Furthermore, our results indicate that genome segments are likely to compete with each other for the available viral proteins and that the balance between different genome segments is affected by reporter genome segment length, by the identity of 3′ and 5′ UTRs, and probably also by their coding regions.

Our results indicate that replication/transcription of a genome segment can be negatively affected by the presence of another genome segment. This interference became less pronounced when the length of the smaller segment was extended, indicating that genome segment length plays a role in the competition between different segments. This “length effect” was also observed when natural genome segments were present in addition to the reporter construct, with the shortest segments, M and NS, giving the strongest inhibition of the reporter gene expression. In agreement herewith, IAV defective interfering (DI) RNAs, which are formed by internal deletion of progenitor RNA segments, interfere with vRNA synthesis, probably because of the competitive advantage of the smaller DI RNA molecules (reviewed by Nayak [29]). In addition, our data indicate that the coding region of the vRNA segment may also be of importance, as the extended version of the *Gaussia* reporter segment was still able to outcompete its firefly luciferase counterpart, albeit less efficiently than its shorter version.

The segment UTRs are known to contain signals for transcription, replication and packaging of vRNP [24], [27], [28], [30]. We now show that the identity of the 3′ and 5′ UTRs also influences the competition between different segments. Relatively minor differences were observed when reporter genome segments with different natural UTRs were compared in the competition assay. This result is in agreement with the observation that non-conserved regions of the UTRs contribute to some but limited extent to viral RNA replication [28], [31]. However, introducing three nucleotide changes in the 3′ UTR (G3A/U5C/C8U) of the NP segment, which is predicted to stabilize the UTR panhandle structure and is known to lead to increased reporter gene expression in infected cells [23], dramatically increased the competitive ability of the reporter segment, both when replication/transcription was driven by transfection of polymerase- and NP-encoding segments and when mediated by IAV infection. Thus, while reporter segments carrying the natural NP UTRs or the mutant NPph UTRs were both efficiently expressed in the absence of competitor segments, large differences in luciferase expression were observed in favor of the luciferase segment carrying the panhandle-stabilizing mutations when other reporter segments were co-transfected or in IAV infected cells. In agreement herewith, recombinant viruses carrying two nucleotide changes (G3A/C8U) in the UTR of either the PB1 or PA segment displayed enhanced replication/transcription of the mutated segments in detriment of the wild-type UTR-bearing segments [32].

The most likely scenario suggested by our observations is that replication/transcription of one reporter segment interferes with that of another by sequestering UTR-binding proteins, probably polymerases, required for RNA synthesis. Several observations by us and others support this hypothesis: 1) increasing the amount of polymerase and NP proteins, but not of NP protein alone, alleviated the competition between different segments, 2) the polymerase proteins have been shown to bind to 5′ and 3′ UTRs of vRNAs, with most strong binding observed to the 5′ UTR [33], 3) introduction of mutations in the 3′ UTR (NPph) that stabilize the panhandle structure and are predicted to result in increased polymerase binding [23] result in increased ability of the reporter segment to be replicated/transcribed in the presence of competitor segments ([23] and this study), 4) introduction of similar mutations in the 5′ UTR (NPphR) that are likely to interfere with polymerase binding [33], had a negative effect on the competitive ability of the reporter construct, and 5) panhandle-stabilizing mutations in the 3′ UTR (NPph), that increased the competitive ability of the reporter construct, partly compensated for replication-debilitating mutations in PB2 (R142A or E361A) [34], [35], but not in NP (M331K or F488G) [36] (Fig. S9), suggesting a link between the interaction of the UTR with polymerase and the ability to compete with other segments.

Although our experimental system (i.e. the transfection assay) does not approach the complexity of the IAV infected cells with respect to number of vRNA segments and viral proteins present, our data suggest that IAV RNA segments compete with each other for available polymerases. This competition is expected to be most apparent early during infection, when only low amounts of polymerase are present. It is conceivable that at this stage of the infection the low level of RNA polymerase plays a critical role in the regulation of segment-specific replication and/or transcription. At later times during infection competition between vRNA segments is expected to be alleviated by the increased levels of the polymerase subunits, thereby ensuring the efficient replication/transcription of all genome segments.

## Materials and Methods

### Cells and Viruses

HEK 293T and MDCK cells were maintained in complete Dulbecco's Modified Eagle's Medium (DMEM; Gibco) containing 10% (v/v) Fetal Calf Serum (FCS; Bodinco B. V.), 100 U/ml Penicillin and 100 µg/ml Streptomycin. Influenza A/WSN/33 (H1N1) (IAV-WSN) was grown on MDCK cells. Briefly ∼70% confluent MDCK cells were infected with IAV-WSN at a multiplicity of infection (MOI) of 0.02 50% tissue culture infectious dose (TCID50) per cell. Supernatant was harvested after 48 h of incubation at 37°C and cell debris was removed by centrifugation at 2,000 rpm for 10 min. Virus was stored at −80°C and TCID50 on MDCK cells was determined.

### Vectors and Vector construction

Precursor firefly and *Gaussia* luciferase reporter gene constructs were generated by GenScript. These constructs have the following schematic make up: hepatitis delta virus (HDV) ribozyme – *AloI* restriction site – firefly or *Gaussia* luciferase gene – *BaeI* restriction site – human PolI promoter. These constructs did not yet contain IAV 5′ and 3′ UTR sequences. 3′ UTR sequences were introduced by ligation of primer dimers into the *AloI*-digested constructs. Subsequently, primer dimers corresponding to 5′ UTR sequences were ligated into the resulting *BaeI*-digested plasmid. The *AloI* and *BaeI* restriction recognition sites are completely removed by this procedure and the corresponding PolI transcripts are similar to genuine vRNA segments with the exception of the coding region. In addition, we generated an extended version of the *Gaussia* luciferase reporter gene construct by introduction of a firefly luciferase gene fragment. As a result, the length of the extended *Gaussia* luciferase vRNA is identical to that of the corresponding firefly luciferase-encoding vRNA. To generate this construct, a *NotI*-digested PCR fragment, corresponding to part of the firefly luciferase gene (nucleotide 559 – stop codon) and containing flanking *NotI* restriction sites, was ligated into the *NotI*-restricted precursor *Gaussia* luciferase plasmid, immediately downstream of the *Gaussia* luciferase gene stop codon. The IAV segment UTRs were inserted in the extended *Gaussia* luciferase plasmid in the same manner as described above. For a schematic overview of the reporter constructs, see Figure 1A.

**Table 2:** Quantitative RT-PCR primers for mRNA of *Gaussia* or firefly luciferase reporter segments flanked by NP or NPph UTRs.

Target	Purpose	Sequence (5′- 3′)
GNP/GNPph	Strand specific primer mRNA	TTTTTTTTTTTTTTTTTTTTTTTAGTCA
	qPCR forward primer	AAGACTTCAACATCGTGGCCG
	qPCR reverse primer	GCAGGTCAGAACACTGCACG
FNP	Strand specific primer mRNA	TTTTTTTTTTTTTTTTTTTTTTTACAAT
	qPCR forward primer	GGATCTACTGGGTTACCTAAGG
	qPCR reverse primer	GGGTTGGTACTAGCAACGCAC

The protein expression plasmids encoding PB2, PB1, PA and NP (pcDNA-PB2, pcDNA-PB1, pcDNA-PA and pcDNA-NP) and transcription plasmids encoding eight IAV-WSN vRNA segments (pPOLI-PB2, pPOLI-PB1, pPOLI-PA, pPOLI-HA, pPOLI-NP, pPOLI-NA, pPOLI-M, and pPOLI-NS) were a kind gift of Dr. Ervin Fodor [37].

### Transfection and infection

HEK 293T cells were seeded in 96-wells plates at a density of 10,000 cells per well and incubated overnight. For the transfection assay, cells were transfected with reporter plasmids encoding firefly or *Gaussia* luciferase along with expression plasmids encoding PB2, PB1, PA, or NP using Lipofectamine 2000 (Invitrogen) according to the manufacturer's protocols. Fifty nanogram of each plasmid was used in the transfection unless mentioned otherwise. For the infection assay, cells were transfected with reporter plasmids encoding firefly or *Gaussia* luciferase. The next day, cells were infected with IAV-WSN at a multiplicity of infection (MOI) of 1 TCID50 units per cells.

### Luciferase Assay

Twenty-four h post-transfection or -infection, cells were lysed by incubation with Passive Lysis Buffer (Promega) for 15 min at room temperature. Cell lysates were assayed for luciferase activity using the Dual-Luciferase assay system (Promega) according to the manufacturer's protocols, and the relative light units (RLU) were determined using a Centro LB 960 Luminometer (Berthold Technologies). The ratio of firefly luciferase/*Gaussia* luciferase activity after co-transfection of both reporter constructs (normFluc/Gluc) was normalized to the ratio of firefly luciferase/*Gaussia* luciferase activity after single transfection of reporter constructs, which was set at 1.

### Influenza A quantitative RT-PCR

Quantitative RT-PCR to determine the amount of mRNA synthesized for the reporter genes during the transfection and infection assays was performed according to Vester et al. [19]. Briefly, following the removal of the cell culture medium, cells were washed with PBS and lysed by incubation with TriZol reagent (Invitrogen) for 3 min at room temperature. The lysates were mixed with chloroform and centrifuged at 14,000 rpm for 20 min at 4°C. The water phase was collected and mixed with 70% (v/v) ethanol. Subsequent RNA purification was performed using the RNAeasy Kit (Qiagen) according to the manufacturer's protocols. The concentration of total RNA was determined using NanoDrop 1000 Spectrophotometer (Thermo Scientific). The total RNA was treated with amplification grade DNase (Invitrogen) according to the manufacturer's protocols to digest the plasmid DNA. Reverse transcription from total RNA was performed using mRNA-specific primer (Table 2). Reverse transcription was carried out using Superscript II reverse transcriptase (Invitrogen). Briefly, 100 ng of DNase-treated total RNA was mixed with 2 pmol of primer and 1 µl of 25 mM dNTP in the total volume of 12 µl. The mixture was incubated at 65°C for 5 min. After the cooling step to 4°C, 4 µl of 5× first strand buffer, 2 µl of 0.1 M DTT, 1 µl of RNase Inhibitor (40 U/µl) were added and the mixture was incubated at 42°C for 2 min. Reverse transcription was carried out at 42°C for 50 min after addition of 1 µl superscript II reverse transcriptase (50 U/µl) and was terminated by heating at 70°C for 15 min. Real time quantitative PCR was performed using qPCR MasterMix Plus for SYBR Green (Eurogentech) on a LightCycler 480 II (Roche). qPCR forward and reverse primers (Table 2) that primed at the coding sequence of corresponding reporter gene were used to amplify cDNA. Quantitative PCR reactions were set up in triplicates according to the manufacturer's instruction by mixing 20 pmol of forward and reverse primers and 1 µl of cDNA products. The PCR mixture was incubated at 95°C for 10 min, followed by 40 cycles of 15 sec and 1 min incubations at 95°C and 60°C, respectively. To check the specificity of PCR product, melting curve analysis was performed at the end of the PCR. The comparative Ct method was used to determine the relative mRNA levels using the housekeeping gene GAPDH as a reference [38], [39]. The mRNA levels were normalized relative to the samples in which a single reporter construct was transfected.

### Statistical analysis

The means of multiple experiments are shown. All experiments were performed 2–4 times, with each experiment containing 4 replicates. Differences between means were determined using Student's t-test. Differences were considered significant if P<0.05. Significant differences are indicated by symbols in the figures where appropriate.

## Supporting Information

Figure S1 **Competition between firefly and** ***Gaussia*** **luciferase reporter genome segments.** Plasmids encoding firefly (FNP) or *Gaussia* (GNP) luciferase reporter constructs were transfected alone (Single) or in combination (Co). Luciferase expression was induced by simultaneous co-transfection of polymerase and NP expression plasmids (transfection assay). A) Firefly luciferase activity after transfection of FNP with empty plasmid (pUC18) or FNP together with GNP. B) *Gaussia* luciferase activity after transfection of GNP with empty plasmid (pUC18) or GNP together with FNP. C) Normalized ratio of firefly to *Gaussia* luciferase activity (Fluc/Gluc) when FNP and GNP were transfected singly or in combination.
(TIF)

Figure S2 **Raw data belonging to** **Figure 2E** **.** A) Firefly luciferase activity. B) *Gaussia* luciferase activity.
(TIF)

Figure S3 **Raw data belonging to** **Figure 3** **.** A and B) Firefly and *Gaussia* luciferase activity belonging to Fig. 3A. C and D) Firefly and *Gaussia* luciferase activity belonging to Fig. 3B. E and F) Firefly and *Gaussia* luciferase activity belonging to Fig. 3C.
(TIF)

Figure S4 **Raw data belonging to** **Figure 4A** **.** A) Firefly luciferase activity. B) *Gaussia* luciferase activity.
(TIF)

Figure S5 **Raw data belonging to** **Figure 4B** **.** A) *Gaussia* luciferase activity. B) Firefly luciferase activity.
(TIF)

Figure S6 **Correlation between firefly luciferase inhibition and** ***Gaussia*** **luciferase expression.** A graph similar to the one shown in Figure 4C, but this time including data obtained with the short *Gaussia* segment (GNP).
(TIF)

Figure S7 **Lack of correlation between genome length and** ***Gaussia*** **luciferase activity increase/decrease.** Correlation between fold-inhibition of *Gaussia* luciferase activity upon co-transfection of one of the eight IAV-WSN vRNA encoding plasmids and the length of the vRNA segments.
(TIF)

Figure S8 **Raw data belonging to** **Figure 6** **.** A and B) Firefly and *Gaussia* luciferase activities belonging to Figure 6B. C and D) Firefly and *Gaussia* luciferase activities belonging to Figure 6C.
(TIF)

Figure S9 **Effect of mutant PB2 and NP on reporter gene expression.** Normalized luciferase activity of firefly (FNP [A] or FNPph [B]) or *Gaussia* (GNP [C] or GNPph [D]) luciferase reporter constructs using the transfection assay in combination with plasmids that encode either wild type PB1, PB2, PA and NP (WT) or mutants thereof. When a plasmid encoding a mutant PB2 (R142A or E361A) or NP (M331K or F488G) was used, instead of the wild type version thereof, this is indicated.
(TIF)
