# Supplementary figures and images for: Competition between Influenza A Virus Genome Segments
Source: PLoS One. 2012 Oct 11;7(10):e47529. doi: 10.1371/journal.pone.0047529 (PMC3469491; doi:10.1371/journal.pone.0047529)

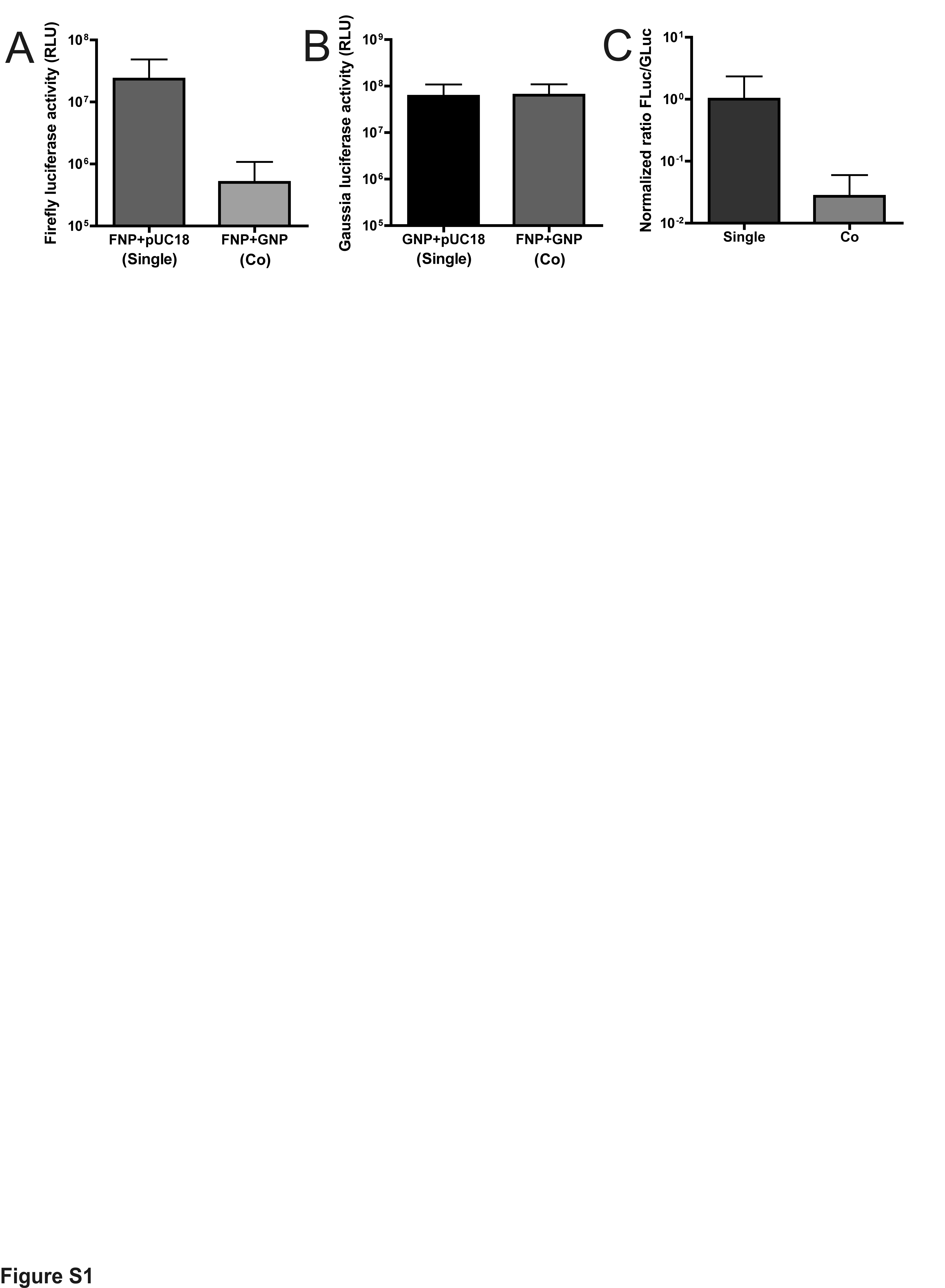

Supplement: Figure S1 — Competition between firefly and Gaussia luciferase reporter genome segments. Plasmids encoding firefly (FNP) or Gaussia (GNP) luciferase reporter constructs were transfected alone (Single) or in combination (Co). Luciferase expression was induced by simultaneous co-transfection of polymerase and NP expression plasmids (transfection assay). A) Firefly luciferase activity after transfection of FNP with empty plasmid (pUC18) or FNP together with GNP. B) Gaussia luciferase activity after transfection of GNP with empty plasmid (pUC18) or GNP together with FNP. C) Normalized ratio of firefly to Gaussia luciferase activity (Fluc/Gluc) when FNP and GNP were transfected singly or in combination. (TIF) [file pone.0047529.s001.tif]

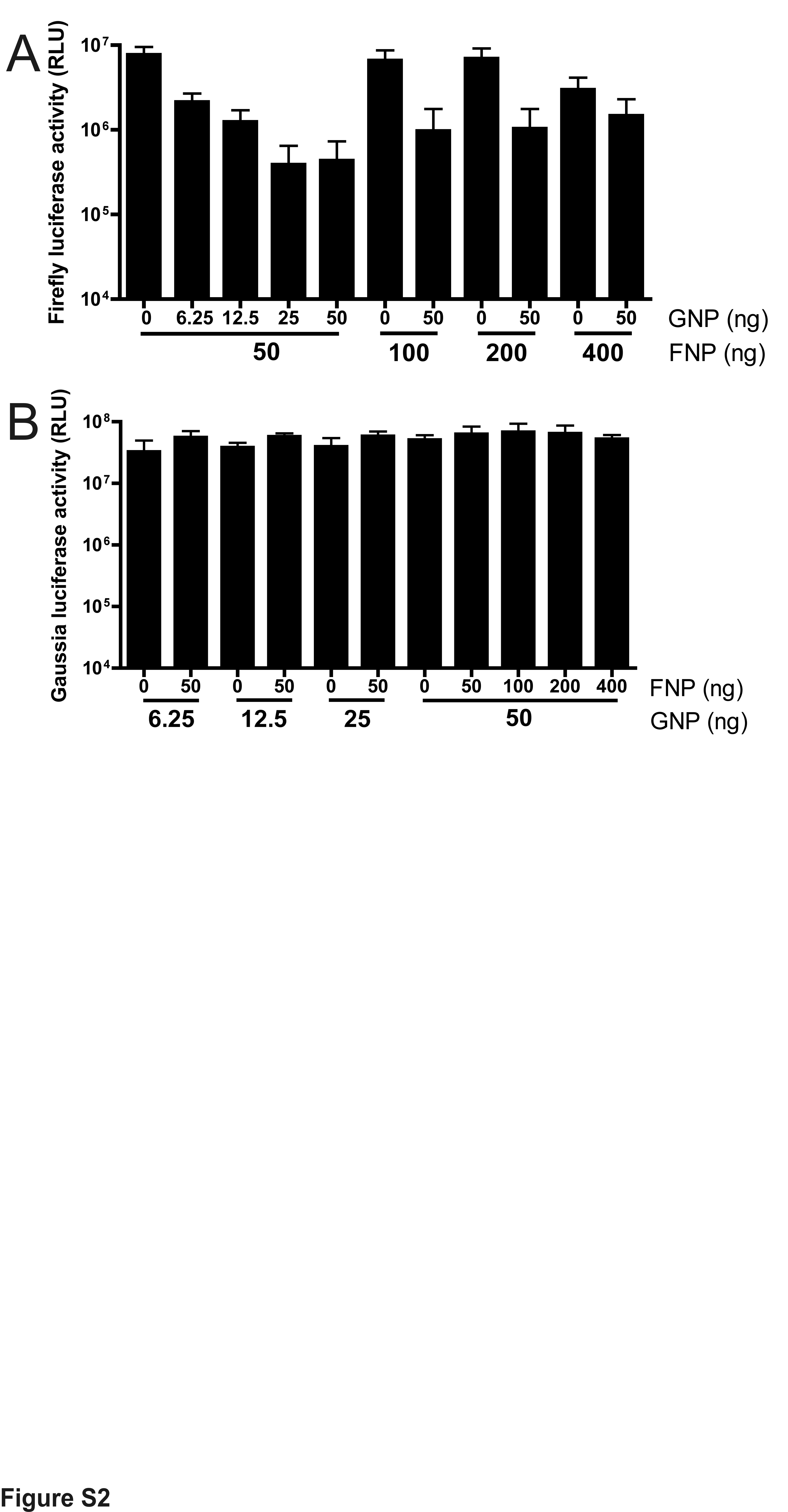

Supplement: Figure S2 — Raw data belonging to Figure 2E . A) Firefly luciferase activity. B) Gaussia luciferase activity. (TIF) [file pone.0047529.s002.tif]

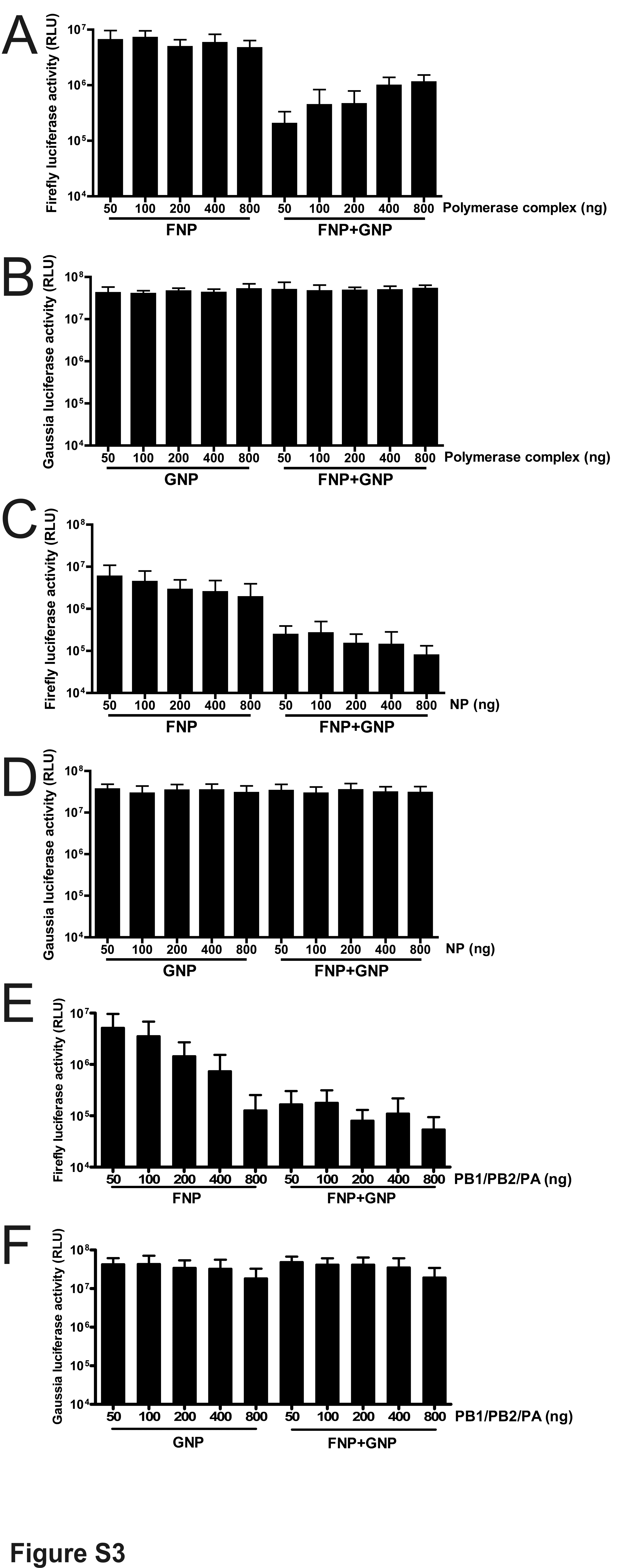

Supplement: Figure S3 — Raw data belonging to Figure 3 . A and B) Firefly and Gaussia luciferase activity belonging to Fig. 3A. C and D) Firefly and Gaussia luciferase activity belonging to Fig. 3B. E and F) Firefly and Gaussia luciferase activity belonging to Fig. 3C. (TIF) [file pone.0047529.s003.tif]

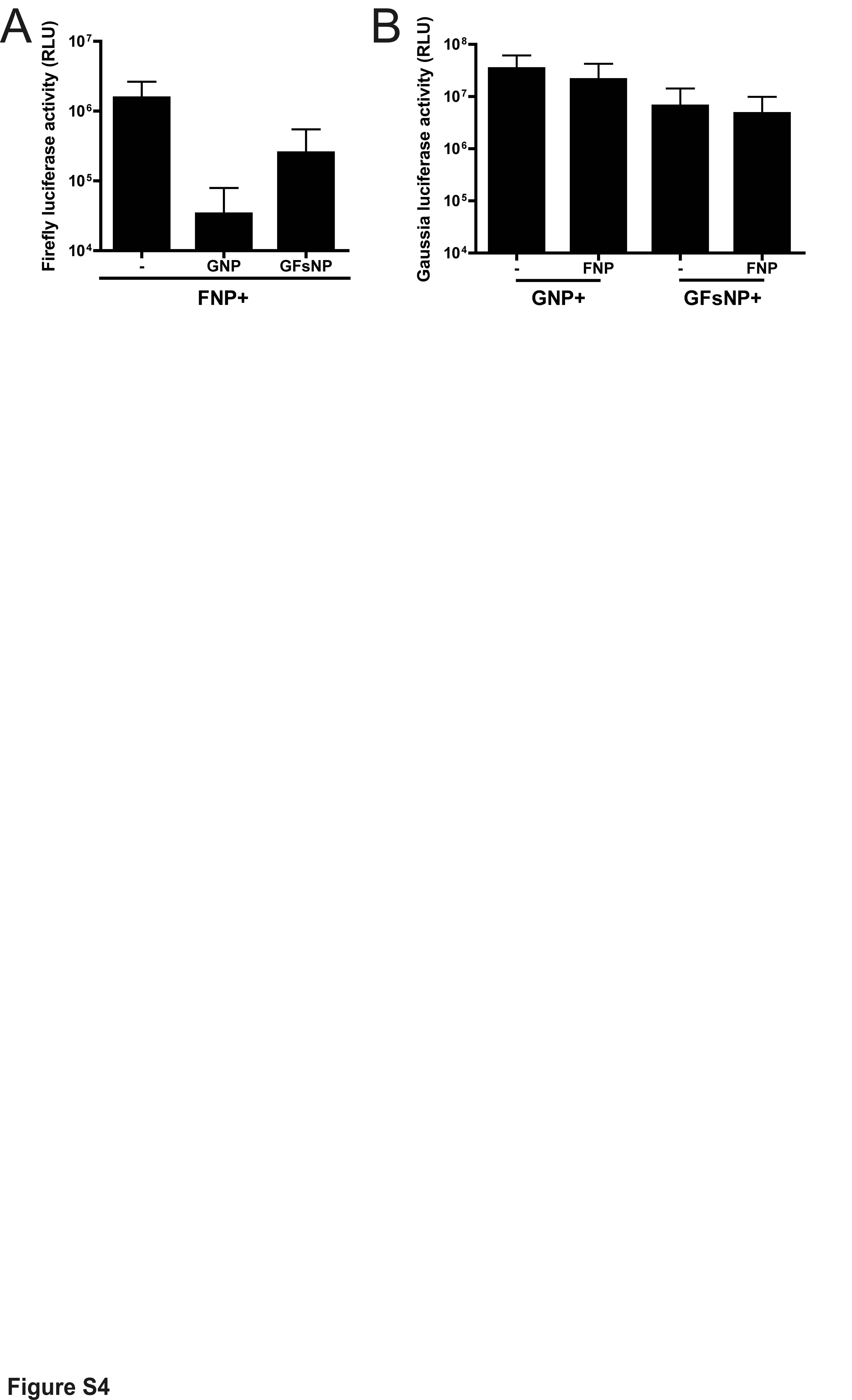

Supplement: Figure S4 — Raw data belonging to Figure 4A . A) Firefly luciferase activity. B) Gaussia luciferase activity. (TIF) [file pone.0047529.s004.tif]

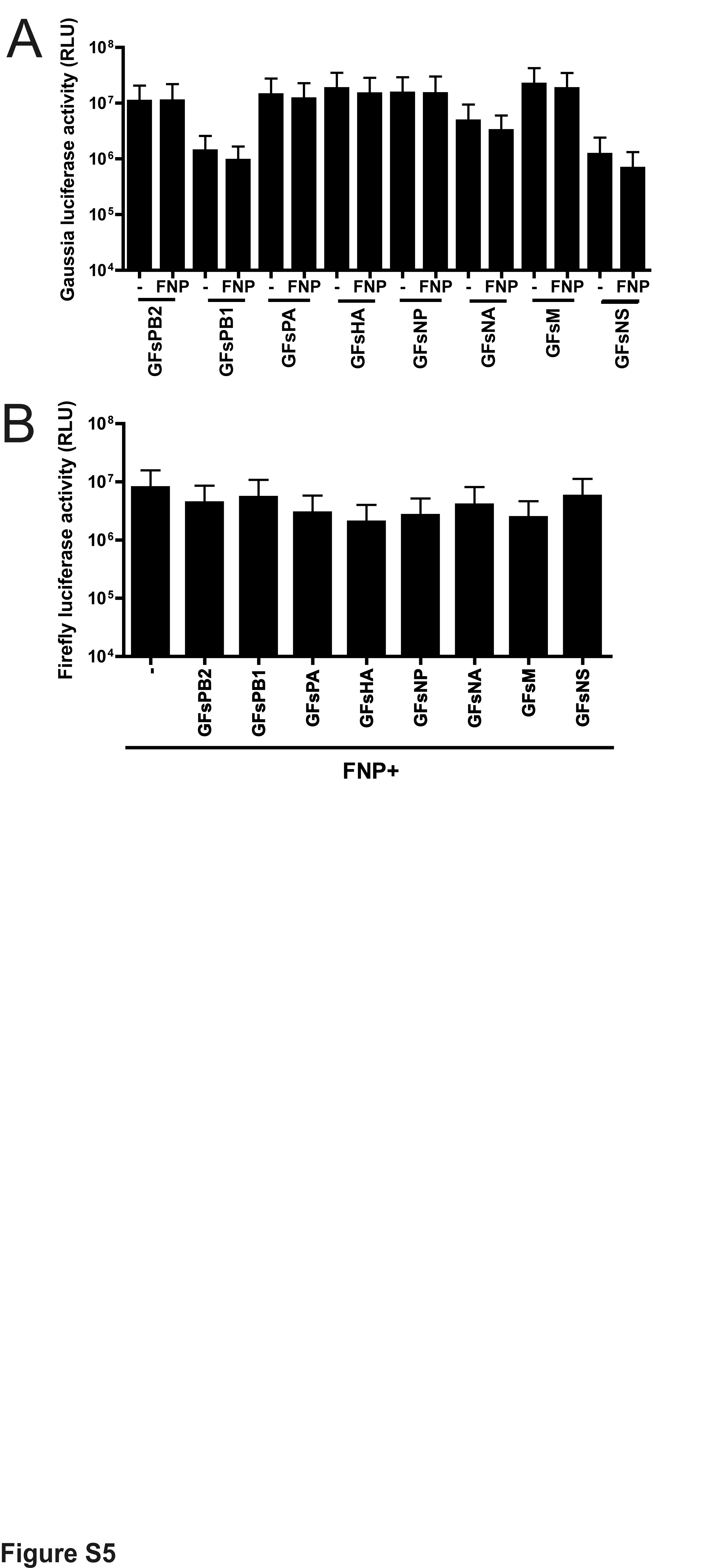

Supplement: Figure S5 — Raw data belonging to Figure 4B . A) Gaussia luciferase activity. B) Firefly luciferase activity. (TIF) [file pone.0047529.s005.tif]

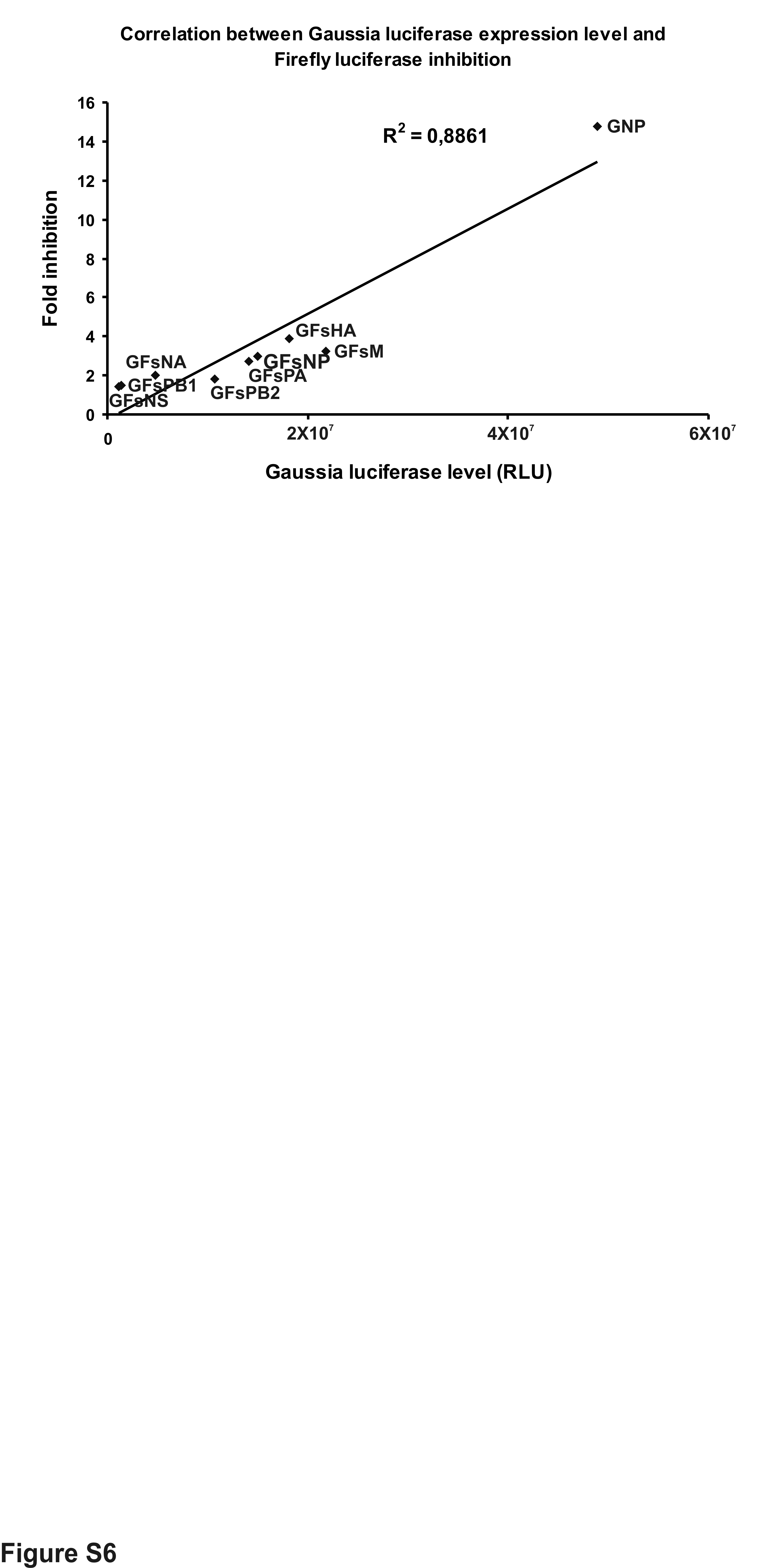

Supplement: Figure S6 — Correlation between firefly luciferase inhibition and Gaussia luciferase expression. A graph similar to the one shown in Figure 4C, but this time including data obtained with the short Gaussia segment (GNP). (TIF) [file pone.0047529.s006.tif]

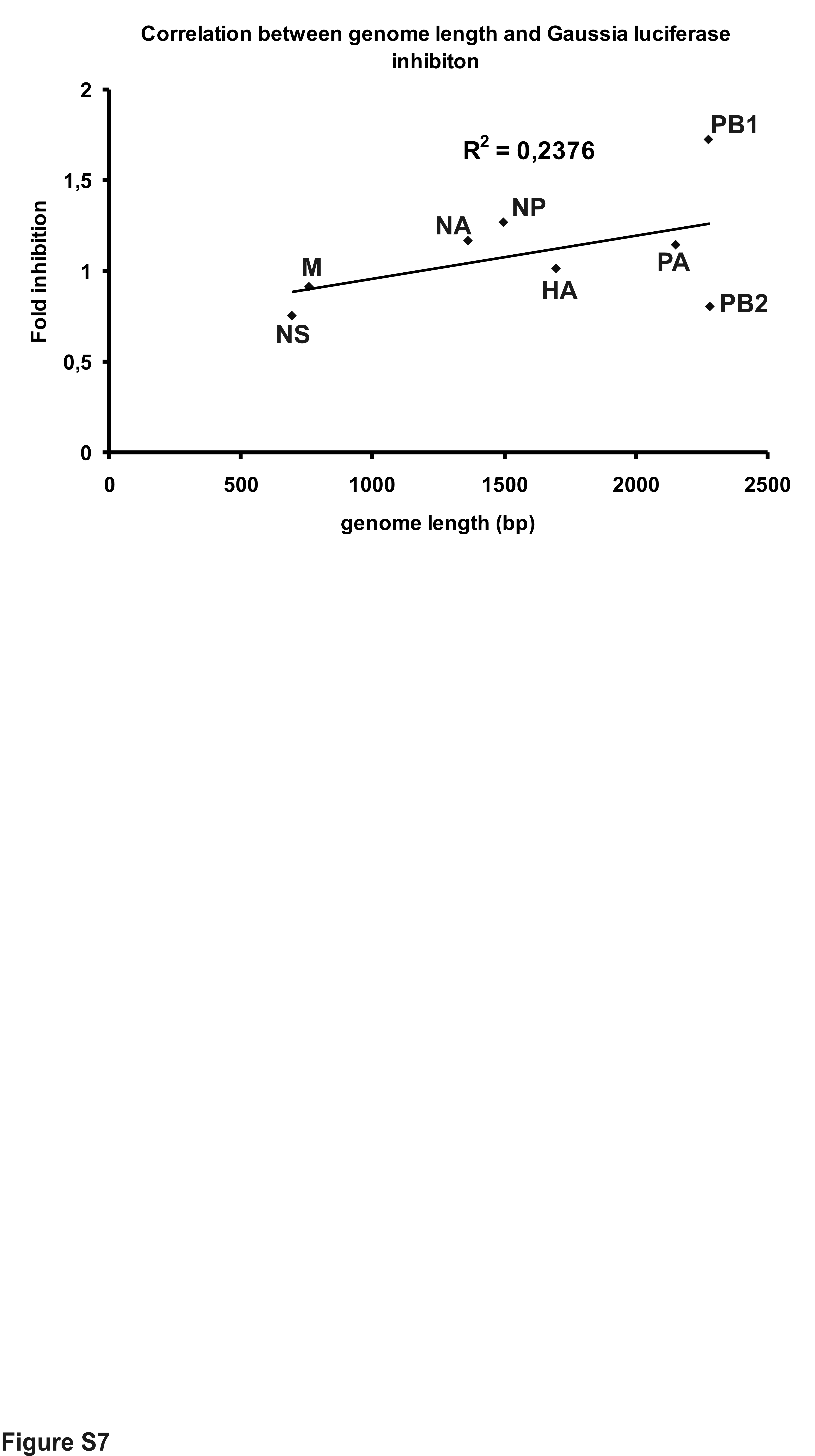

Supplement: Figure S7 — Lack of correlation between genome length and Gaussia luciferase activity increase/decrease. Correlation between fold-inhibition of Gaussia luciferase activity upon co-transfection of one of the eight IAV-WSN vRNA encoding plasmids and the length of the vRNA segments. (TIF) [file pone.0047529.s007.tif]

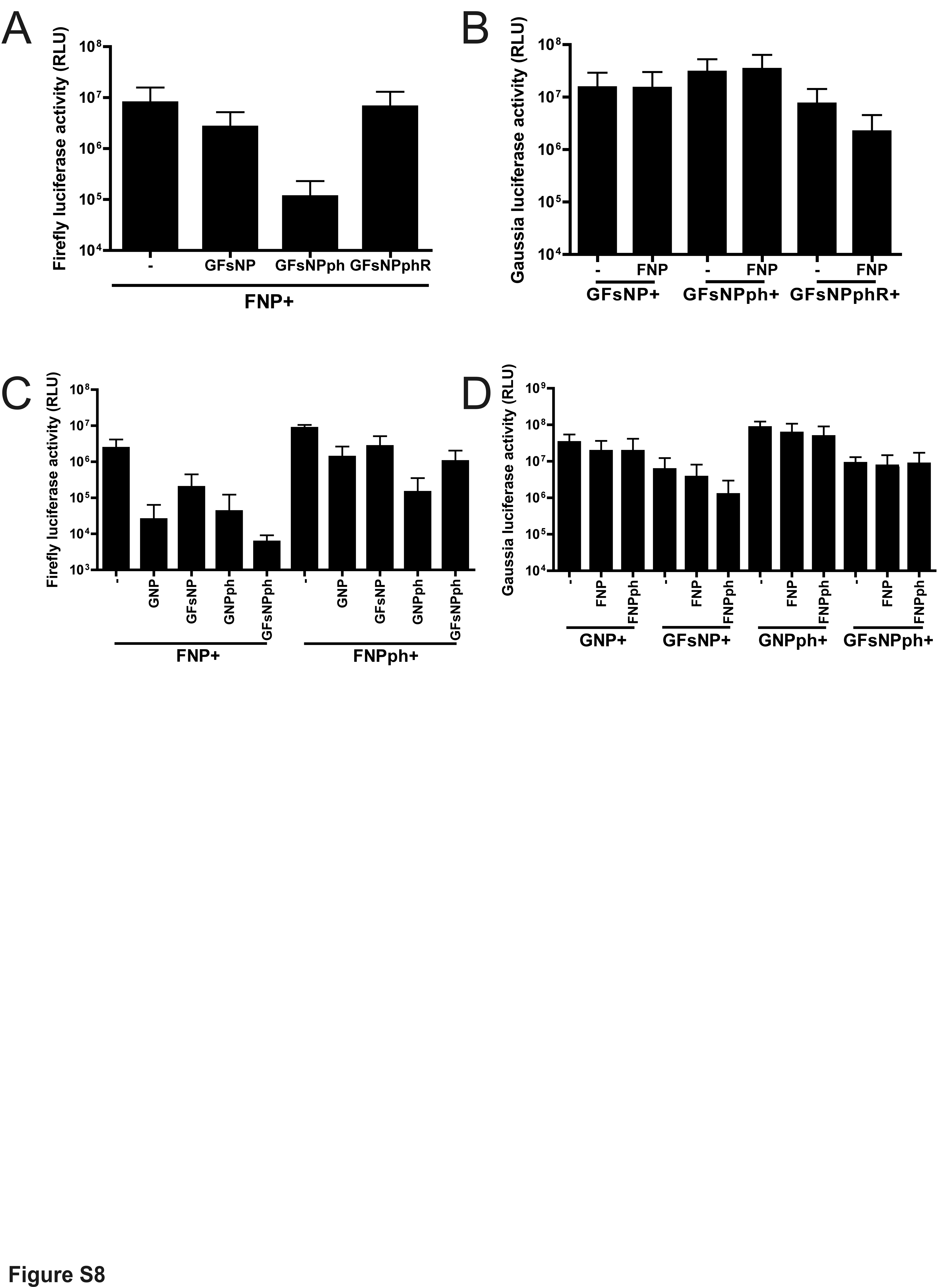

Supplement: Figure S8 — Raw data belonging to Figure 6 . A and B) Firefly and Gaussia luciferase activities belonging to Figure 6B. C and D) Firefly and Gaussia luciferase activities belonging to Figure 6C. (TIF) [file pone.0047529.s008.tif]

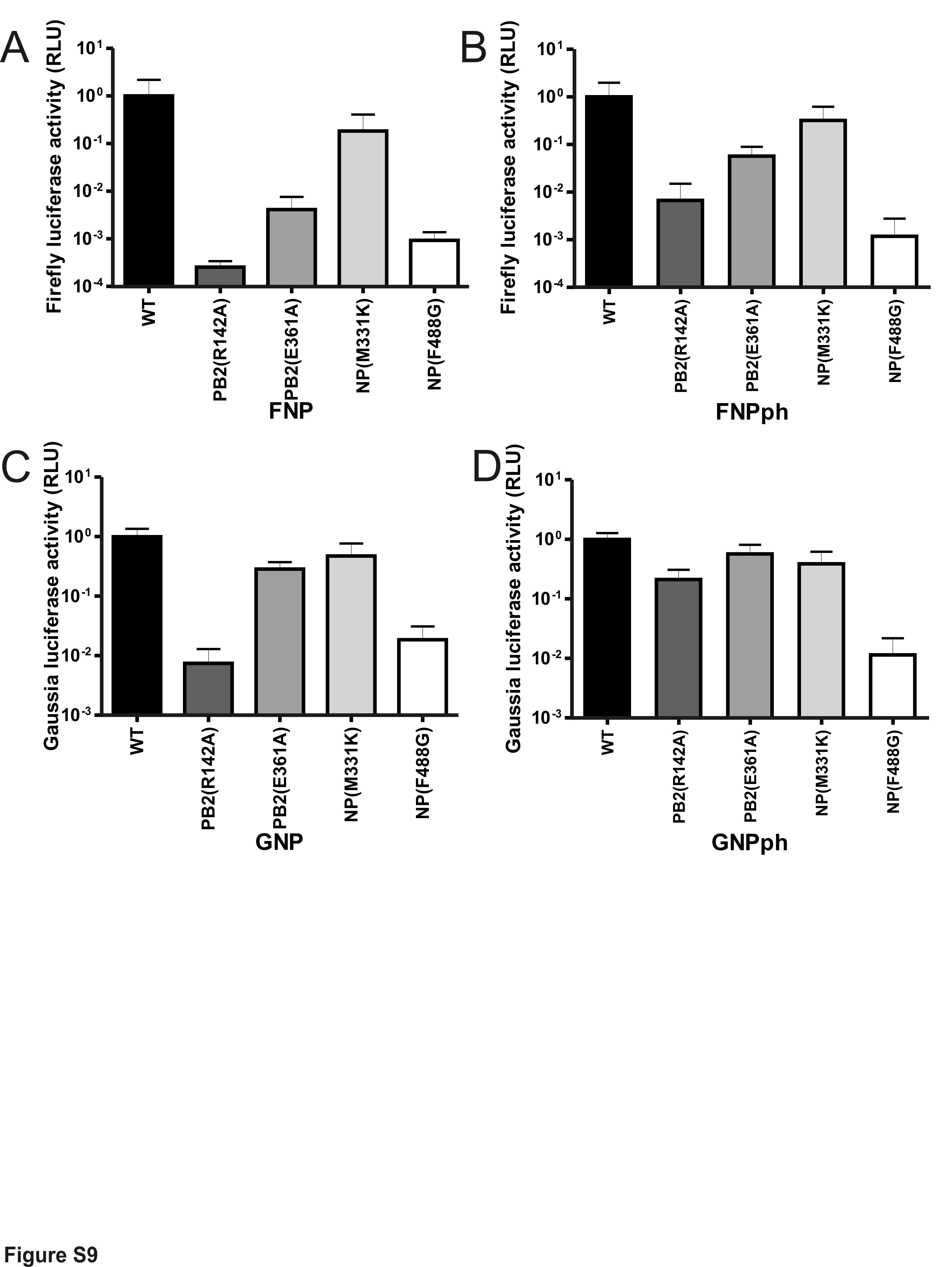

Supplement: Figure S9 — Effect of mutant PB2 and NP on reporter gene expression. Normalized luciferase activity of firefly (FNP [A] or FNPph [B]) or Gaussia (GNP [C] or GNPph [D]) luciferase reporter constructs using the transfection assay in combination with plasmids that encode either wild type PB1, PB2, PA and NP (WT) or mutants thereof. When a plasmid encoding a mutant PB2 (R142A or E361A) or NP (M331K or F488G) was used, instead of the wild type version thereof, this is indicated. (TIF) [file pone.0047529.s009.tif]
